# Multi-View Ensemble Classification of Brain Connectivity Images for Neurodegeneration Type Discrimination

**DOI:** 10.1007/s12021-017-9324-2

**Published:** 2017-02-16

**Authors:** Michele Fratello, Giuseppina Caiazzo, Francesca Trojsi, Antonio Russo, Gioacchino Tedeschi, Roberto Tagliaferri, Fabrizio Esposito

**Affiliations:** 1 Department of Medical, Surgical, Neurological, Metabolic and Aging Sciences, Second University of Naples, Naples, Italy; 2 Department of Medicine Surgery and Dentistry Scuola Medica Salernitana, University of Salerno, Baronissi, Salerno, Italy; 3 Department of Medicine, Surgery and Dentistry “Scuola Medica Salernitana”, University of Salerno, Via S. Allende, 84081, Baronissi, Salerno, Italy

**Keywords:** Multi-view, Multi-modality, Random forests, Amyotrophic lateral sclerosis, Parkinson’s disease, Fractional anisotropy, Default mode network

## Abstract

Brain connectivity analyses using voxels as features are not robust enough for single-patient classification because of the inter-subject anatomical and functional variability. To construct more robust features, voxels can be aggregated into clusters that are maximally coherent across subjects. Moreover, combining multi-modal neuroimaging and multi-view data integration techniques allows generating multiple independent connectivity features for the same patient. Structural and functional connectivity features were extracted from multi-modal MRI images with a clustering technique, and used for the multi-view classification of different phenotypes of neurodegeneration by an ensemble learning method (random forest). Two different multi-view models (intermediate and late data integration) were trained on, and tested for the classification of, individual whole-brain default-mode network (DMN) and fractional anisotropy (FA) maps, from 41 amyotrophic lateral sclerosis (ALS) patients, 37 Parkinson’s disease (PD) patients and 43 healthy control (HC) subjects. Both multi-view data models exhibited ensemble classification accuracies significantly above chance. In ALS patients, multi-view models exhibited the best performances (intermediate: 82.9%, late: 80.5% correct classification) and were more discriminative than each single-view model. In PD patients and controls, multi-view models’ performances were lower (PD: 59.5%, 62.2%; HC: 56.8%, 59.1%) but higher than at least one single-view model. Training the models only on patients, produced more than 85% patients correctly discriminated as ALS or PD type and maximal performances for multi-view models. These results highlight the potentials of mining complementary information from the integration of multiple data views in the classification of connectivity patterns from multi-modal brain images in the study of neurodegenerative diseases.

## Introduction

In Machine Learning applications, using different independent data sets (e.g. from different measurement modalities) to represent the same observational entity (e.g. a patient in a clinical study), is sometimes referred to as multi-view (MV) learning (Sun 2013). Assuming that each “view” encodes different, but potentially complementary, information, an MV analysis would treat each single view (SV) data set with its own statistical and topological structures while attempting to classify or discriminate the original entities on the basis of both data views.

Functional and anatomical brain connectivity studies are providing invaluable information for understanding neurological conditions and neurodegeneration in humans (Agosta et al. 2013; Chen et al. 2015). In clinical neuroimaging based on multi-modal magnetic resonance imaging (MRI), functional connectivity information can be extracted from blood oxygen level dependent (BOLD) functional MRI (fMRI) time-series, usually acquired with the patient in a resting state (rs-fMRI), whereas anatomical connectivity information is typically obtained from the same patient using diffusion tensor imaging (DTI) or similar techniques applied to diffusion-weighted MRI (dMRI) time-series (Sui et al. 2014; Zhu et al. 2014). Thereby, addressing connectivity and neurodegeneration from both data types can be naturally framed within the same MV analysis of MRI images (Hanbo Chen et al. 2013).

Functional and anatomical connectivity analyses can be performed using either voxel- or region-of-interest (ROI) based methods applied to the available fMRI and dMRI data sets. The voxel space is the native space of both image types and therefore retains the maximum amount of spatial information about whole-brain connectivity; however this information is spread over tens of thousands (in 3 Tesla MRI) or millions (in 7 Tesla MRI) spatial dimensions. After functional pre-processing, one or more parametric maps can be calculated to represent connectivity information at each voxel. Fractional anisotropy (FA) maps, obtained from DTI data sets via tensor eigenvalue decomposition (Basser and Jones 2002), and default-mode network (DMN) component maps, obtained from rs-fMRI data sets via independent component analysis (ICA) or seed-based correlation analyses (van den Heuvel and Pol 2010), have been the most commonly employed images in structural and functional clinical studies of brain connectivity.

ICA decomposition values from rs-fMRI do not describe the functional connectivity between two specific brain regions. Similarly, FA values from DTI modelling of dw-MRI do not describe the structural connectivity between two specific regions. Nonetheless, in many research and clinical applications, ICA values are used to describe the spatial distribution (over the whole brain) of certain rs-fMRI signal components that fluctuate coherently in time within a given functional brain network (van de Ven et al. 2004; Beckmann et al. 2005; Ma et al. 2007). In the absence of systematic task-related activations, as in the case of the resting state, both the amount of synchronization of rs-fMRI fluctuations and their spatial organization as functional networks, is fundamentally due to functional connectivity processes, thereby the ICA values are considered spatially continuous descriptors of functional connectivity effects which are not constrained to a pre-specified number of regions.

In contrast to voxel-based methods, in the so called connectome approaches (Sporns et al. 2005), a dramatically lower number of regions, usually up to one or two hundreds, is predefined using standard atlas templates or known functional network layouts, and region-to-region fMRI-derived time-course correlations and dMRI-reconstructed fibre tracts are calculated, yielding a graph model of brain connectivity (Sporns 2011). An MV clustering technique has been previously proposed in the context of graph theoretic models to derive stable modules of functional and anatomical connectivity across healthy subjects (Hanbo Chen et al. 2013). However, while the dramatically reduced spatial dimensionality allows highly detailed and complex connectivity models to be estimated according to brain physiology and graph theory (Fornito et al. 2013), the a priori definition of “seed” ROIs may sometimes excessively constrain, and potentially dissolve (part of), the information content of the input images. Moreover, the use of the same set of regions to constrain both fMRI and dMRI data sets may introduce some sort of dependence between the views. On the other hand, using individual voxels as features is usually considered not robust enough for individual connectivity pattern classification and discrimination. In fact, both the extremely high dimensionality of intrinsically noisy data sets like the fMRI and dMRI maps and the inter-subject anatomical and functional variability of the voxel-level connectivity maps easily make the statistical learning highly sensible to errors (Flandin et al. 2002).

To alleviate both the curse of dimensionality and the problem of misaligned and noisy voxels, here we propose to use the approach of *feature agglomeration* (Thirion et al. 2006; Jenatton et al. 2011) in the context of voxel-based MV connectivity image analysis. In this approach, the whole brain volume is partitioned into compact sets of voxels (i.e. clusters) that jointly change as coherently as possible across subjects. In combination with agglomerative clustering in the voxel space, an ensemble learning technique called Random Forests (RF) (Breiman 2001) is applied to the MV neuroimaging data sets. Due to its non-linear and multivariate nature, the RF has been previously shown to best capture important effects in MV data sets, and to improve prediction accuracy in the context of MV learning (Gray et al. 2013). There are three common strategies to define MV data models: early, intermediate and late integration (Pavlidis et al. 2001). Early integration is performed by concatenating the features of all views prior to further processing; intermediate integration defines a new joint feature space created by the combination of all single views; late integration aggregates the predictions derived by models trained on each single view.

Using individual pre-calculated DMN and FA maps from independently acquired 3 Tesla rs-fMRI and DTI-dMRI data sets, we applied the intermediate and late MV integration approaches for the RF-based MV learning, to the problem of classifying age-matching elderly subjects as belonging to one out of three different classes: Amyotrophic Lateral Sclerosis (ALS) patients, Parkinson’s Disease (PD) patients and healthy controls (HC).

Both ALS and PD are neurodegenerative diseases that progressively impair the ability of a patient to respectively start or smoothly perform voluntary movements; however, they are extremely different for what concerns the pathological mechanism. In fact, while ALS affects motor neurons (progressively leading to their death), PD affects dopamine-producing cells in the substantia nigra, causing a progressive loss of movement control. The majority (i.e. about 90%) of all ALS and PD cases are of sporadic type, meaning that the cause is unknown (de Lau and Breteler 2006; Kiernan et al. 2011).

For both diseases, diagnosis is performed by experienced neurologists with a series of standard clinical tests that basically exclude other pathologies with similar behaviour. However, both PD and ALS generally exhibit highly variable clinical presentations and phenotypes and this makes the diagnosis and patient classification challenging. In particular, there is no definitive diagnostic test for ALS, which is sometimes identified on the basis of both clinical and neurophysiologic signs (Brooks et al. 2000; de Carvalho et al. 2008).

According to recent epidemiological data, the diagnosis rate of PD (Hirsch et al. 2016) is 2.94 and 3.59 (new cases per 100,000 persons per year, respectively for females and males) in the age range of 40–49 years, reaches the peaks of 104.99 and 132.72 in the range of 70–79 years and drops to 66.02 and 110.48 in the range of 80+ years. For ALS (Logroscino et al. 2010), the diagnosis rate is definitely lower: 1.5 and 2.2 in the range of 45–49 years, 7.0 and 7.7 in the range of 70–79 years and 4.0 and 7.4 in the range of 80+ years. This suggests that the development of reliable diagnostic and prognostic biomarkers would represent a significant advance, especially in the clinical work-up of ALS.

Previous neuroimaging studies have demonstrated that ALS and PD can be better characterized by taking into account multiple measurement types (Douaud et al. 2011; Aquino et al. 2014; Foerster et al. 2014). Here, the complementary information encoded in DMN and FA views has been exploited for the SV and MV RF classification of ALS and PD patients as well as of healthy controls.

## Methods

### Ethics Statement

The institutional review board for human subject research at the Second University of Naples approved the study and all subjects gave written informed consent before the start of the experiments.

### Participants

We acquired data from 121 age-matched subjects ranging from *38* to *82* years of age (mean age *63.87 ± 8.2*). These included *37* (*14* women and *23* men) patients with a diagnosis of PD according to the clinical diagnostic criteria of the United Kingdom Parkinson’s disease Society Brain Bank, *41* ALS patients (*20* women and *21* men) fulfilling the diagnostic criteria for probable or definite ALS, according to the revised El Escorial criteria of the World Federation of Neurology (Brooks et al. 2000) and *43* volunteers (*23* women and *20* men).

### MRI Data Acquisition and Pre-Processing

MRI images were acquired on a 3 T scanner equipped with an 8-channel parallel head coil (General Electric Healthcare, Milwaukee, Wisconsin).

DTI was performed using a repeated spin-echo echo planar diffusion-weighted imaging sequence (repetition time = 10,000 ms, echo time = 88 ms, field of view =320 mm, isotropic resolution =2.5 mm, b value =1000 s/mm^2^, 32 isotropically distributed gradients, frequency encoding RL). Rs-fMRI data consisted of 240 volumes of a repeated gradient-echo echo planar imaging T2*-weighted sequence (TR = 1508 ms, axial slices =29, matrix =64 × 64, field of view =256 mm, thickness = 4 mm, inter-slice gap =0 mm). During the scans, subjects were asked to simply stay motionless, awake, and relax, and to keep their eyes closed. No visual or auditory stimuli were presented at any time during functional scanning.

Three-dimensional T1-weighted sagittal images (GE sequence IR-FSPGR, TR = 6988 ms, TI = 1100 ms, TE = 3.9 ms, flip angle =10, voxel size =1 mm × 1 mm × 1.2 mm) were acquired in the same session to have high-resolution spatial references for registration and normalization of the functional images.

DTI data sets were processed with the FMRIB FSL (RRID:SCR_002823) software package (Jenkinson et al. 2012). Pre-processing included eddy current and motion correction and brain-tissue extraction. After pre-processing, DTI images were concatenated into 33 (1 B = 0 + 32 B = 1000) volumes and a diffusion tensor model was fitted at each voxel, generating the FA maps.

Rs-fMRI data were pre-processed with the software BrainVoyager QX (RRID:SCR_013057, Brain Innovation BV, Maastricht, the Netherlands). Pre-processing included the correction for slice scan timing acquisition, the 3D rigid body motion correction and the application of a temporal high-pass filter with cut-off set to three cycles per time course. From each data set, 40 independent components (ICs), corresponding to one sixth of the number of time points (Greicius et al. 2007) and accounting for more than 99.9% of the total variance, were extracted using the plug-in of BrainVoyager QX implementing the fastICA algorithm (Hyvarinen 1999). To select the IC component associated with the DMN, we used a DMN spatial template from a previous study on the same MRI scanner with the same protocol and pre-processing (Esposito et al. 2010). The DMN template consisted of an inclusive binary mask obtained from the mean DMN map of a separate population of control subjects and was here applied to each single-subject IC, in such a way to select the best-fitting whole-brain component map as the one with the highest goodness of fit values (GOF = mean IC value inside mask – mean IC value outside mask) (Greicius et al. 2004, 2007). To avoid ICA sign ambiguity, each component sign was adjusted in such a way to have all GOF positive-valued.

Both diffusion and functional data were registered to structural images, and then spatially normalized to the Talairach standard space using a 12-parameter affine transformation. During this procedure, the functional and diffusion images were all resampled to an isometric 3 mm grid covering the entire Talairach box. After spatial normalization, all resampled EPI volumes were visually inspected to assess the impact of geometric distortion on the final images, which was judged negligible given the purpose of analysing whole-brain distributed parametric maps rather than regionally specific effects.

### Overview of the Methodology

**Fig. 1:**
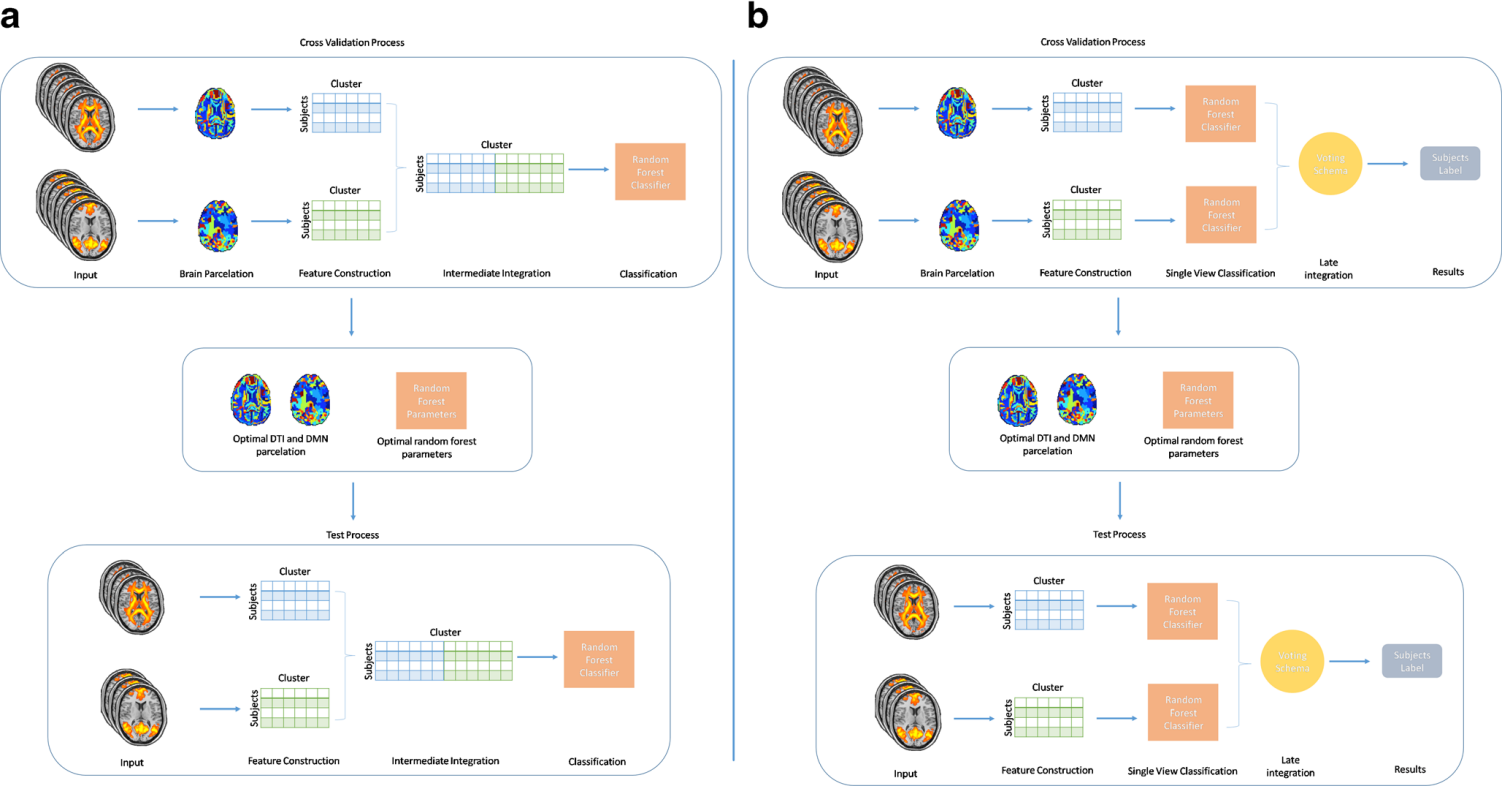
**a** Intermediate Data integration model. Preprocessed input images are parcelated by unsupervised clustering. The parcelation is used to compute the features that are concatenated and used to train the MV intermediate integration RF model. The training procedure is performed in nested cross-validation and the resulting best parameters are used to estimate the generalization capability of the model on the held-out fold. **b** Late Data integration model. Preprocessed input images are parcelated using by unsupervised clustering. The obtained parcelation is used to compute the features that are used to train the SV RFs. The resulting classifications are integrated to generate the MV prediction. The training procedure is performed in nested cross-validation and the best parameters are used to estimate the generalization capability of the model on the held-out fold

The proposed approaches are schematically represented in Fig. 1. After preprocessing, each view dimensionality is independently reduced by a hierarchical procedure of voxel agglomeration (“Feature Agglomeration” section). We applied the additional constraint that only adjacent areas can be merged in order to get contiguous brain areas. Each brain area is then compressed in a robust feature computing the median of the corresponding voxel values for each subject. The features are then used to train the two MV classification algorithms (“Random Forest Classifier” section).

Following the distinction made in (Pavlidis et al. 2001), the proposed models belong to the following two categories:

- *Late Integration*
  Two independent RFs are trained on functional and structural feature sets. The MV prediction is based on a majority vote approach made according to the classification results of the forests from each single view. This is done by merging the sets of trees from the SV RFs and counting the predictions obtained by this pooled set of trees. This method has the advantage of being easily implemented in parallel, since each model is trained on a view independently from the other but it does not take into account the interactions that may exist between the views.

- *Intermediate Integration*
  Data is integrated during the learning phase. For this purpose, an intermediate composite dataset is created by concatenating the features of each view. This approach has the advantage of learning potential inter-view interactions. As a downside, a larger number of parameters must be estimated, and additional computational resources are necessary.

### Feature Agglomeration

Brain activity and brain structural properties are usually spread over an area bigger than the volume of a single voxel. Aggregating adjacent voxels together improves signal stability across subjects, while reducing the number of features, and may translate in improved prediction capabilities.

We built a common data driven parcelation of the brain by clustering the voxels across all the subjects. The clustering was unsupervised and performed once for all subjects of each training dataset, resulting in one common parcelation for each single view. This produced the single-view features that are (eventually) concatenated for the intermediate integration (see Fig. 1). As the clustering operates in the space of subjects, the features are simply concatenated along the subject dimension, thereby the correspondence of each cluster across subjects is preserved.

Voxels are aggregated using hierarchical agglomerative clustering with the Ward’s criterion of minimum variance (Ward 1963). The clustering procedure is further constrained by allowing only adjacent voxels to be merged. This procedure allowed a data-driven parcelation yielding a new set of features (clusters of voxels) that corresponded to brain areas of arbitrary shape that were maximally coherent across training subjects. This methodology of construction of higher level features has been used in (Jenatton et al. 2011) and (Michel et al. 2012). In (Jenatton et al. 2011) the authors used the hierarchical structure derived from the parcelation to regularize two supervised models trained on both synthetic and real-world data. Previous works already showed that, compared to standard models, these regularized models yield comparable or better accuracy, and that the maps derived from the weights exhibit a compact structure of the resulting regions. In (Michel et al. 2012), the parcelation was derived from the hierarchical clustering in a supervised manner, i.e., by explicitly maximizing the prediction accuracy of a model trained on the corresponding features. Although this procedure is not guaranteed to converge to an optimum, experimental results on both synthetic and real data showed a very good accuracy.

### Decision Tree Classifier

**Fig. 2:**
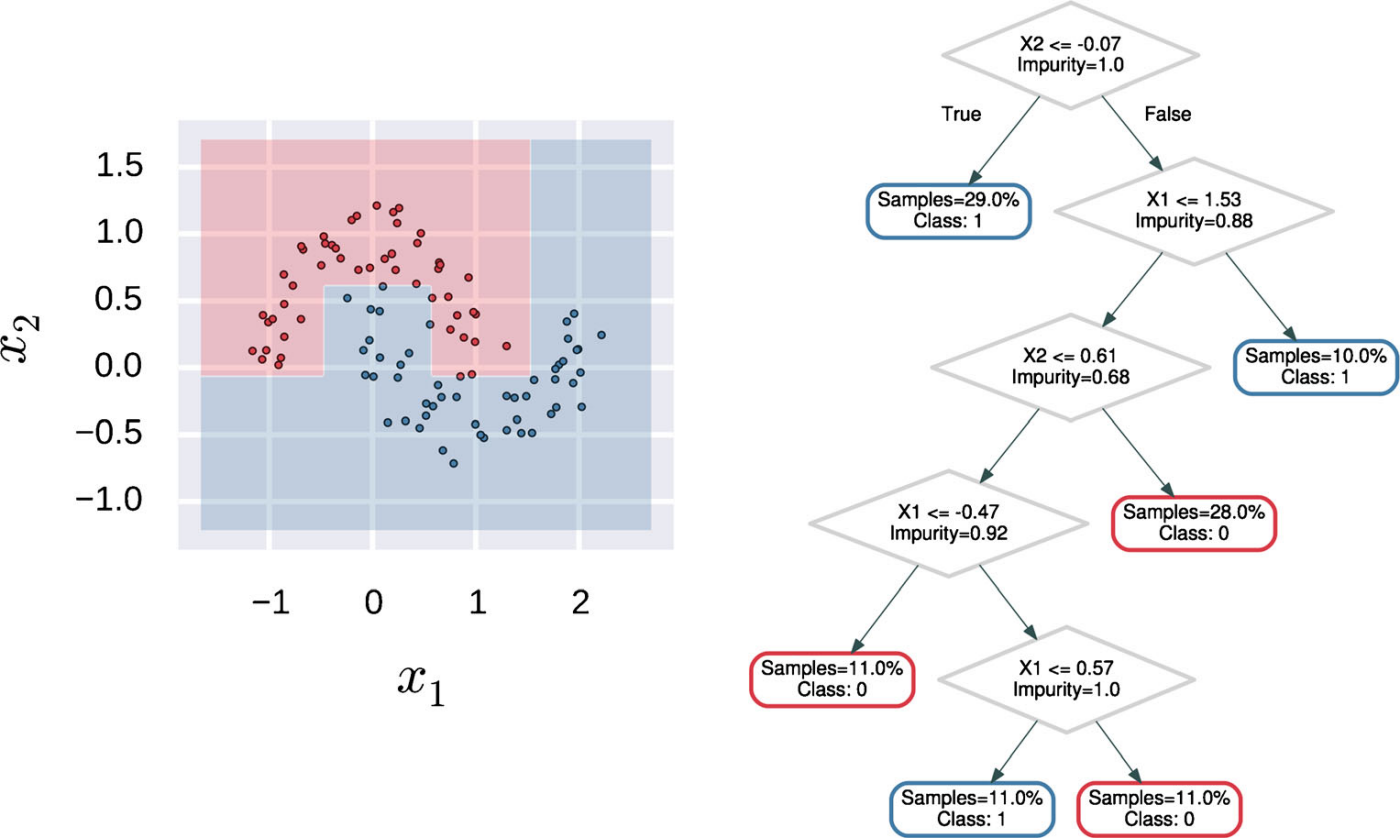
A decision tree with its decision boundary. Each node of the decision tree represents a portion of the feature space (left). For each data point, its predicted class is obtained by visiting the tree and evaluating the rules of each inner node. When a leaf node is reached, then the corresponding class is returned as the prediction (right)

Decision tree classifiers produce predictions by splitting the feature space into axis-aligned boxes were each partitioning increases a criterion of purity (Fig. 2). The most common purity indices for classification are:

$$ \mathrm{Cross}-\mathrm{Entropy}:-\sum_{k=1}^K{\widehat{p}}_k\mathit{\log}\left({\widehat{p}}_k\right) $$

$$ \mathrm{Gini}\ \mathrm{Index}:\sum_{k=1}^K{\widehat{p}}_k\left(1-{\widehat{p}}_k\right) $$

Where ${\widehat{p}}_k$ is the proportion of samples of each class *k* associated to a given node (Hastie et al. 2009).

The main advantages of decision trees are the low bias in prediction and the high interpretability of the model. Despite their simplicity, decision trees are flexible enough to capture the main structures of data. On the other hand, decision trees are highly variable, meaning that small variations in the training data can produce different partitioning of the feature space, and hence unstable predictions.

### Random Forest Classifier

An RF is an *ensemble* method based on *bagging* (bootstrap aggregating) (Breiman 1996). A large set of potentially unstable (i.e. possibly with a high variance in predictions) but *independent* classifiers are aggregated to produce a more accurate classification with respect to each single model. Here, with classification independence, we mean that the labels predicted from different classifiers are as much uncorrelated as possible across the observations. One of the few requirements for ensemble methods to work is that the single classifiers in the ensemble have accuracy better than chance. In fact, even an accuracy slightly higher than chance would be sufficient to guarantee that the probability that the whole ensemble predicts the wrong class is exponentially reduced. The full independency of the classifiers is needed to ensure that possible wrong predictions are rejected by the rest of correct classifiers which are expected to be higher in number, thereby increasing the overall accuracy (Dietterich 2000).

The base predictor structure used in RF is the decision tree, hence the name.

Random forests handle multi-class problems without the need of transformation heuristics, like One-vs-One or One-vs-Rest which are necessary to extend binary classifiers like SVMs to multi-class classification problems and which suffer from potential ambiguities (Bishop 2006).

Independency of the predictors is ensured by training each predictor on a bootstrapped training dataset and randomly sampling a subset of features each time a splitting of the dataset has to be estimated (Breiman 2001).

Training an RF consists in training an ensemble of decision trees: each decision tree is trained on a bootstrapped dataset, i.e., sampled with replacement from the original dataset and with the same dimensionality.

Each sample in the original dataset has a probability of ${\left(1-\frac{1}{N}\right)}^N$ of not appearing in a bootstrapped dataset. Particularly, this probability tends to $\frac{1}{e}\approx 0.3679$ for *N* → ∞, where *N* is the number of samples in the original dataset. This means that each decision tree is trained on a bootstrapped dataset that, on average, has roughly two thirds of samples of the original dataset plus some replicated samples. The remaining one third of samples in the original dataset not appearing in the bootstrapped dataset is used to estimate the generalization performance of the tree. These generalization estimates are aggregated into the Out Of Bag (OOB) error estimate of the ensemble. Through the OOB error, it is possible to estimate the generalization capabilities of the ensemble without the need of an hold-out test set (Breiman 2001). Empirical studies showed that the OOB error is as accurate in predicting the generalization accuracy as using a hold-out test set, or a cross-validation scheme when data is not sufficiently abundant, given a sufficient number of estimators in the forest to make the OOB estimate stable (Breiman 1996).

However, since we perform a feature clustering procedure before training the forest we cannot exploit OOB estimates but rely on cross-validation. This is because voxel agglomeration is performed before RF training, meaning that if a train/test split is defined after the agglomeration (as would be in the case of bootstrapping the training dataset for each tree in the forest) some information about the test data of each tree gets passed into the partitioning, potentially leading to over-optimistic biases in the estimate of generalization performances.

We also evaluated for each feature, the average measure of improvement in the purity criterion each time a feature is selected for a split as an index of the relevance of that feature to the classification.

### Model Settings and Classification

Prior to training the models, the effect of age and sex is removed from the voxels via linear regression. We performed this operation at the voxel level to avoid that the obtained parcelation could encode age or sex similarities rather than functional and/or structural similarities across subjects.

**Fig. 3:**
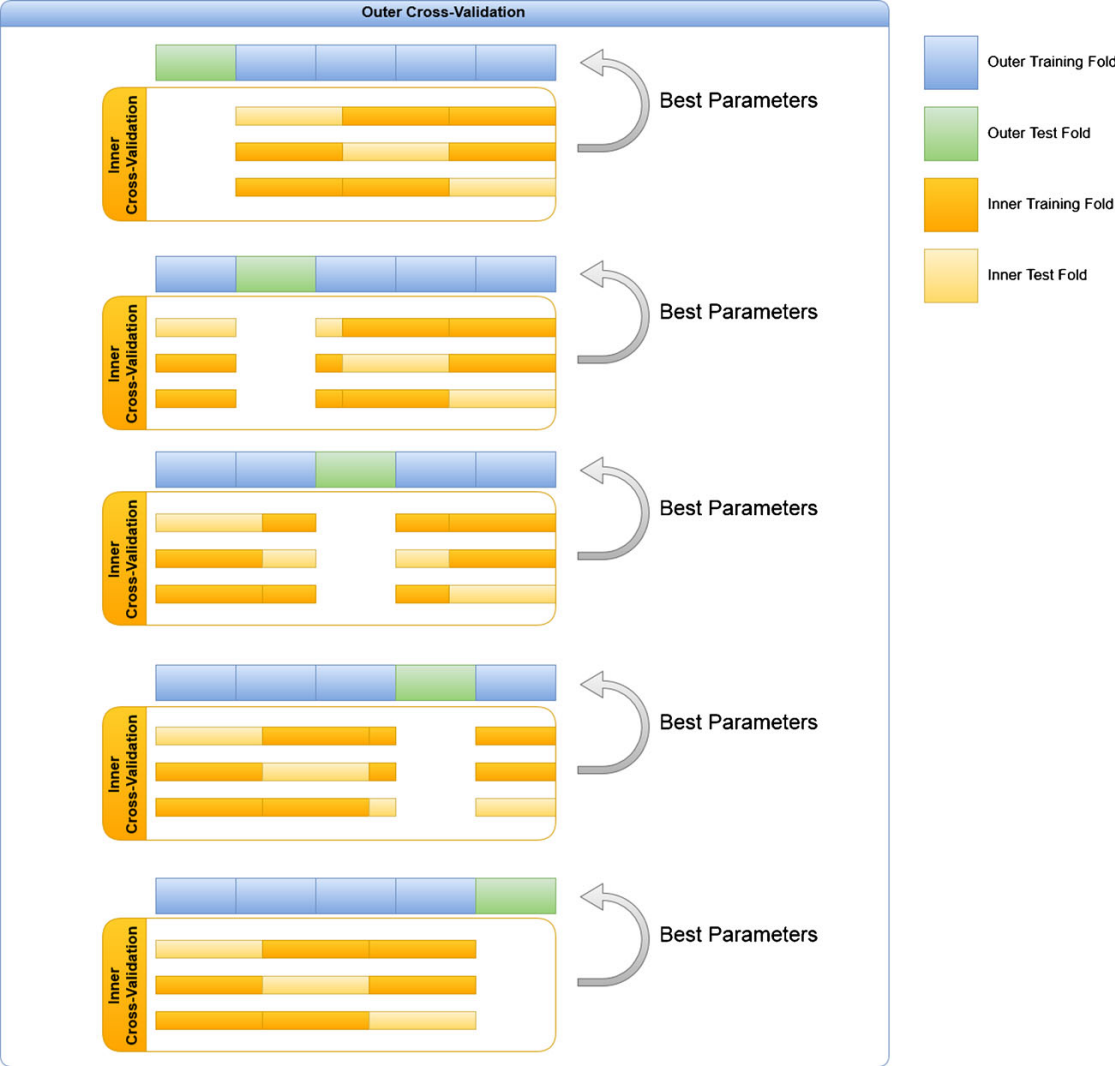
Training schedule used for each SV and MV model. The data is recursively partitioned into outer and inner training and test sets by a nested cross-validation scheme. The inner train/test splits are used to estimate the best parameters configurations, whereas the outer train/test splits are used to estimate the generalization capabilities of the models trained with the best performing configurations of parameters

Each SV and MV model is trained with two nested cross-validation loops. After preprocessing, the whole dataset is partitioned into 5 outer disjoint subsets of subjects (or folds). Iteratively, all subjects of one outer fold are set aside and only used as test subjects to estimate the generalization performances of the model. All subjects belonging to the remaining 4 outer folds are used to estimate the best configuration of parameters (number of clusters, features, number of trees, impurity criterion) and to train the models. To optimize parameters, all subjects belonging to the 4 outer folds were further partitioned into 3 inner folds (nested loop cross-validation). In the inner loop, 2 out of the 3 inner folds are used to train the models by varying the parameter configuration and the third (held-out) inner fold is used to estimate the accuracy performance of that configuration. The accuracies for each parameter configuration are averaged across the held-out inner folds and the best performing configuration of parameters is used to train each model on all the data of the 4 outer folds. The models trained with the best parameters are then tested on the held-out outer fold and the results across the held-out outer folds are averaged to estimate the generalization performances for each model. This training scheme is graphically represented in Fig. 3. The same operations were also repeated by permuting the labels of the train subjects in the outer folds to estimate the null distribution (see “Performance Evaluation” section).

For each training set, the entire brain volume is parcelled in an unsupervised manner using the clustering obtained from the different views.

The features resulting from the unsupervised step are used to train two types of MV classifiers depending on whether the integration is performed before or after the training of RF (intermediate and late integration, respectively).

In each model, the actual number of brain areas (clusters) had to be chosen as a trade-off between the compactness of a cluster in the subject space (i.e. coherence across subjects) and its size (number of voxels).

### Performance Evaluation

The generalization performances of the best parameter configurations of each model estimated by nested cross-validation were assessed by permutation testing. We built the empirical null hypothesis by training *500* classifiers for each model where we first permuted the samples’ labels and then collected the accuracies.

To further investigate the performances of the proposed models in the classification of healthy controls, we defined the following assessment procedure: for each healthy control *x*_*c*_ in our dataset, we trained each proposed model *100* times by randomly choosing *70%* of the dataset as the training. To rule out the possibility that the resulting models would be over trained, we assessed the quality of the predictions of each of these models by evaluating their predictions on the corresponding *30%* hold-out data not used for training. We also ensured that the training set did not contain *x*_*c*_ and recorded its predicted class labels. We repeated this experiment twice: in the former, the training set comprised the HCs, whereas in the latter the classifiers were trained only on the pathologic classes. In this way, it was possible to verify whether, and quantify to what extent, the possible wrong assignment of a given healthy control was driven by a specific selection of the training examples, or, rather, by a systematic bias (i.e. the features of some of the healthy controls would effectively result more similar to those of the ALS or PD patients than to those of the other controls). Particularly, we expect that the majority HCs correctly recognized have unstable predictions in the case of classifiers trained only on pathologic classes. On the other hand, stable but wrong predictions in the case of classifiers trained with HCs, should be somewhat reflected or amplified in the case of training without HCs.

We also generated brain maps of feature relevance. For each model, a brain area (cluster) was assigned a score depending on how much, on average, a split on that feature reduces the impurity criterion. A high score corresponds to high impurity reduction, i.e. the feature is more important. These scores were normalized such that the sum of all importance values equals to 1 in each view. In order to make the scores from different models anatomically comparable, we assigned the score of each brain cluster to all the corresponding voxel members, normalized by the number of voxels that form the region. Normalization ensures that the sum of the scores across all voxels still sums to 1. Thus, the resulting score maps have the same scales for all models and can be compared across models.

## Results

### Brain Parcelation

Using a simple gaussian model (see, e. g., Forman et al. 1995), we preliminary estimated the mean spatial smoothness of each individual functional and structural map prior to running the feature agglomeration procedure. These calculations yielded a mean estimated smoothness of 2.16 +/− 0.47 voxels for the DMN maps and of 2 +/− 0.23 voxels for the DTI maps. We used these maps (without spatial smoothing) to obtain the brain parcelation.

As we observed that (across the folds) different numbers of parcels for DMN and DTI resulted in optimal performances (reported in Table 1), we decided to choose the configurations that contain a number of clusters equal to *500* for both DMN and DTI, thus allowing the majority of cluster sizes to range from *10* to *150* voxels, which represents a good compromise considering the typical cluster sizes found for regional effects in neuroimaging.

**Table 1:** Accuracies of the proposed models compared to the respective null hypothesis

Model	Chance accuracy	Estimated accuracy	p-value
Single-View (DMN)	0.354 ± 0.094	0.650 ± 0.078	<10^−6^
Single-View (FA)	0.322 ± 0.098	0.582 ± 0.118	<10^−6^
Multi-View (Intermediate)	0.351 ± 0.091	0.667 ± 0.150	<10^−6^
Multi-View(Late)	0.342 ± 0.091	0.675 ± 0.141	<10^−6^

This choice produced a new dataset for each view made of *500* features derived from the clustering. In the case of late integration, each single view model was fitted to single dataset of dimensionality *121* subjects × *500* features, whereas in Intermediate Integration we used a merged dataset of *121* subjects × *1000* features.

### Random Forest Parameters

For each ensemble model, we assessed the number of trees, the purity criterion and the number of features to sample when estimating the best split.

In the case of late integration, at least *10,000* trees were necessary to reach the maximum generalization on the outer cross-validation. For the intermediate integration, at least *15,000* trees were necessary.

For both integration strategies, results with the Entropy purity criterion were slightly better compared to the Gini index.

Lastly, in both models, the number of randomly selected features for splitting had little or no influence on the accuracy estimates, thereby we chose to set it to $\sqrt{p}$ as suggested in (Breiman 2001), where *p* is the number of features.

### Performances

**Fig. 4:**
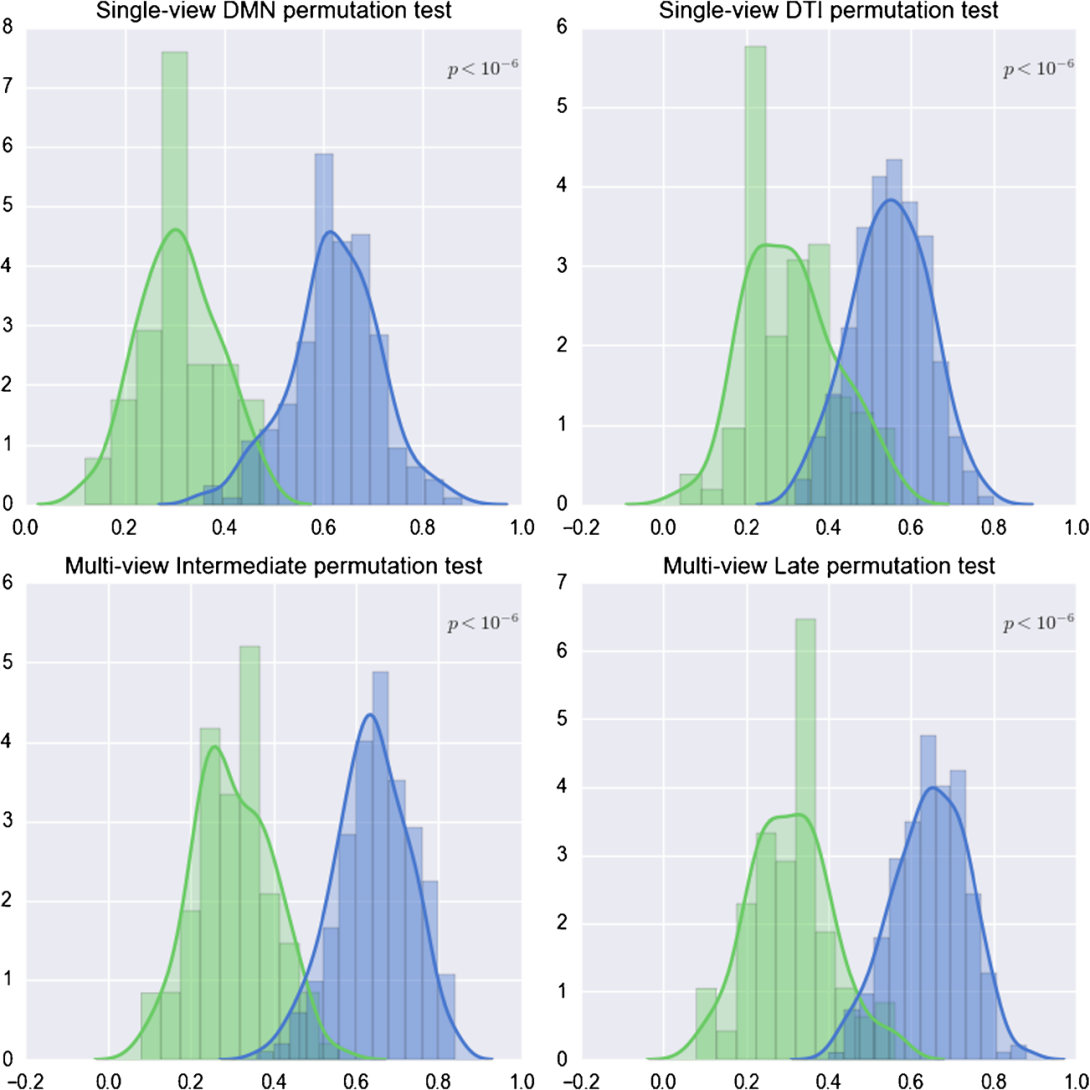
Distribution of the generalization accuracies (blue histograms) estimated for each SV and MV model. The null distribution of the generalization accuracy (green histograms) is computed by permuting the labels of the dataset and repeating the training 500 times for each model to obtain the significance of the statistical test

Performance evaluations for both SV and MV models are illustrated in Fig. 4, where the null distributions of the estimated accuracies are shown together with the corresponding non-permuted case. For all models, the classification accuracies were significantly higher than those obtained under the null hypothesis (see Table 1), that can be rejected with high statistical confidence (*p* < 10^−6^).

**Fig. 5:**
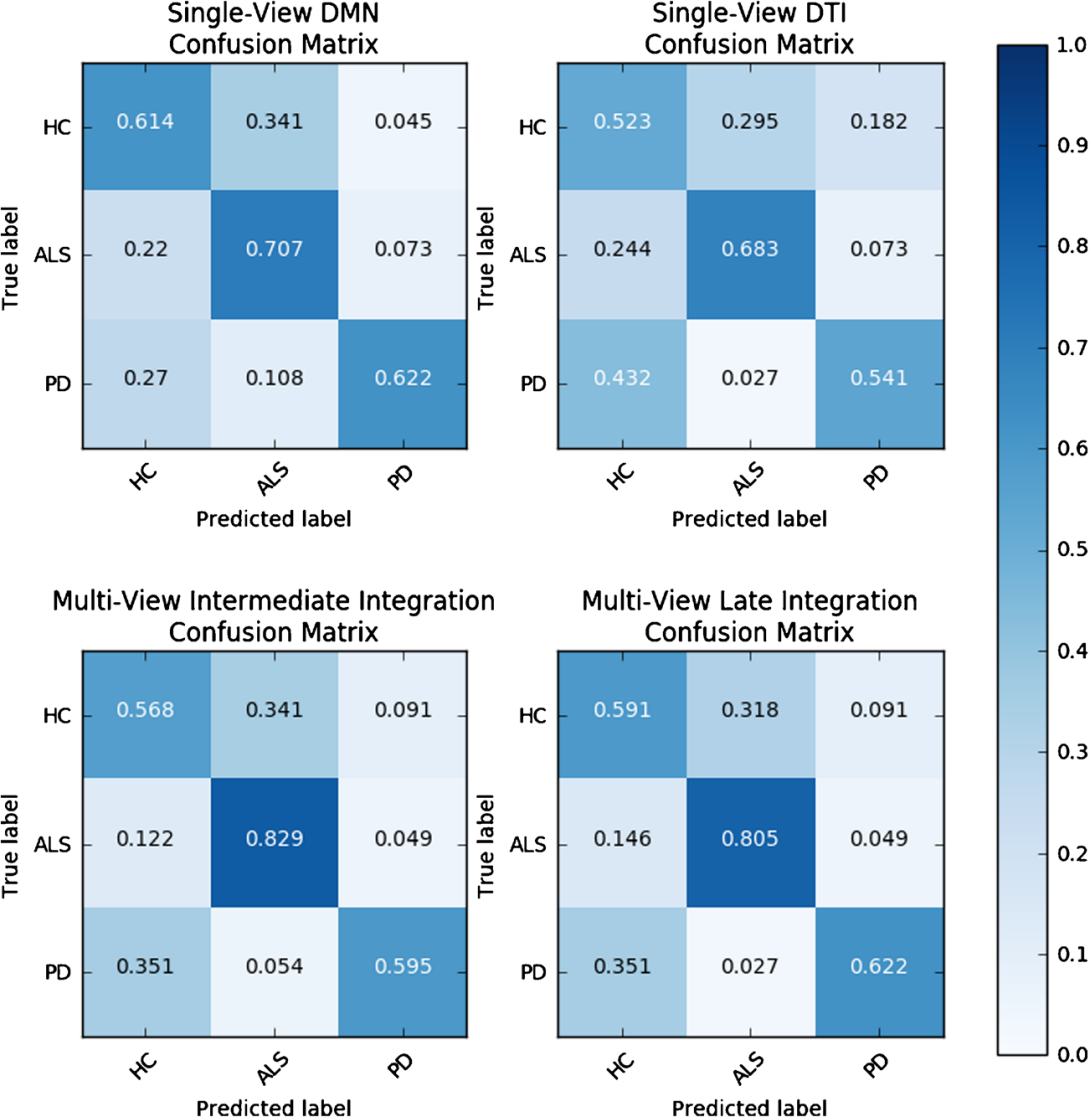
Class-specific accuracies computed for each SV and MV model reported as confusion matrices. Each row reports the percent of subjects belonging to each class, whereas each column corresponds to the percent of subjects belonging to a predicted class

The classifier confusion matrices (i.e. the accuracies reported for each class) for all models are reported in Fig. 5 and show that the performances are not homogenous across classes. Generally, the models’ discrimination capability is higher when it comes to distinguish among pathologies compared to the discrimination between pathology and healthy conditions. The SV model trained only on DMN maps has better classification accuracy for ALS patients (*70.7%*) compared to PD patients (*62.2%*) and HC (*61.4%*). The SV model trained only on FA maps has better classification accuracy for ALS patients (*68.3%*) compared to PD patients (*54.1%*) or HC (*52.3%*). MV classifiers have better classification accuracy for ALS patients, reaching *82.9%* for Intermediate and *80.5%* for Late. PD patient classification accuracy after integration is on the other hand comparable to the SV models, with Intermediate integration reaching *59.5%* and Late Integration reaching *62.2%*. In both MV models, HC classification accuracy is slightly degraded with respect to the best SV model, scoring *56.8%* in Intermediate Integration and *59.1%* in Late Integration.

**Fig. 6:**
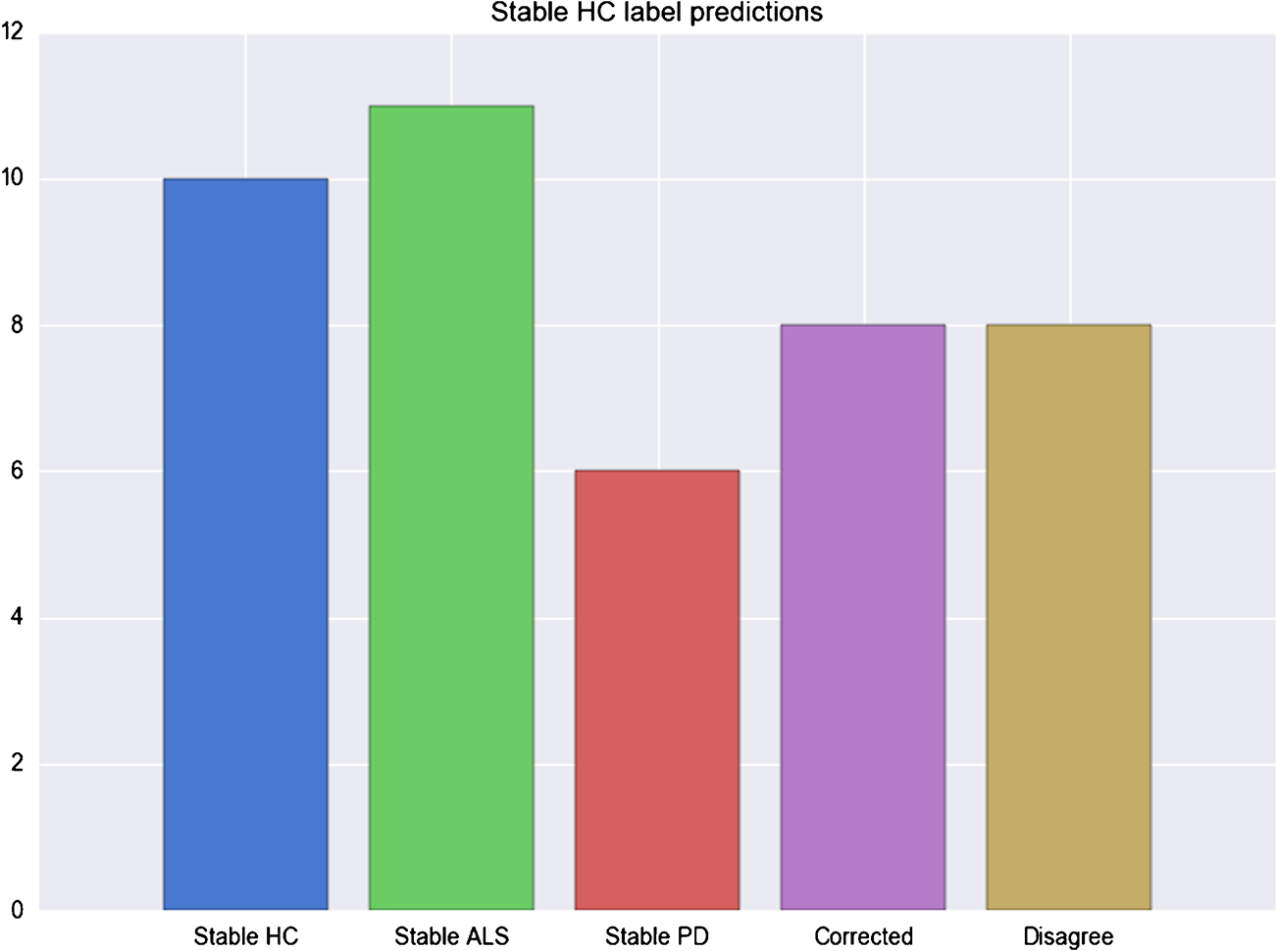
Stable label predictions for HC subjects partitioned based on the behaviour of the predicted labels computed by sampling 100 different training sets for each HC

When repeating the training process keeping each HC outside the training set, we considered as the final class label of each HC the majority label across all the *100* classifiers for each data integration type. We identified five groups, shown in Fig. 6, in which controls can be separated depending on the predictions obtained by each SV and MV model: (i) a group of 10 HC that are systematically classified with the correct label by each SV and MV model; (ii) a group of 11 HC that are consistently classified by both SV and MV models as ALS; (iii) a group of 6 HC that are consistently classified as PD by each SV and MV model; (iv) a group of 8 HC that are classified correctly as controls by at most one SV model and get the correct label by MV models; (v) a group of 8 HC for which the predictions among the SV are in disagreement resulting in unstable MV predictions.

In the case of training on pathologic classes only, HCs of group (i) were split into *5* controls with a stable classification as PD, 1 control classified as stable ALS and 4 controls for which the SV and MV models are in disagreement. HC classified with a stable label as ALS (group ii) or PD (group iii) maintain their stable labels also in this case. Similarly for group (i), the HC which are correctly classified only by MV models (group iv) are split into a single HC with a stable ALS prediction, 4 HC with a stable PD prediction and 3 HC for which predictions are unstable. Finally, the HC of group (v) for which there was disagreement among views in the 3-class scenario are partitioned into 3 HC with stable ALS label, 3 HC with stable PD label and 2 HC with unstable prediction.

Albeit not surprising, we noted that, when trained only with pathological classes, the accuracies of the classifiers considerably increase. In fact, ALS accuracy reaches the highest value of 92.3% for the SV DMN classifier, while both the intermediate and late MV classifiers reached 93.8%, whereas the accuracy of the SV DTI classifier reaches an accuracy of 83.6%. For PD patients, the highest accuracy is reached by the MV late classifier (86.9%) compared to the SV DMN classifier and the MV intermediate (both reaching 84.2%). Also in this case, the SV DTI classifier achieves a slightly lower accuracy of 79%.

**Fig. 7:**
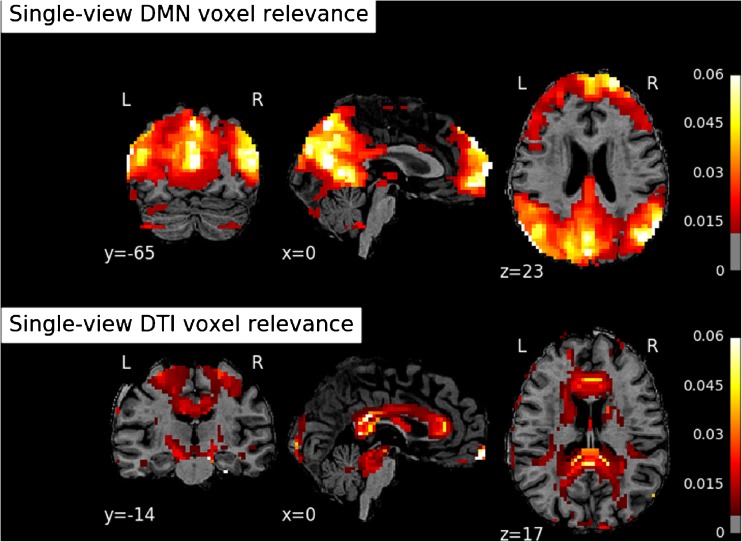
Voxel cluster relevance maps (triplanar reslicing: coronal, sagittal, transversal) computed from the single-view Random Forests. Upper row: DMN view. Bottom row: FA view

**Fig. 8:**
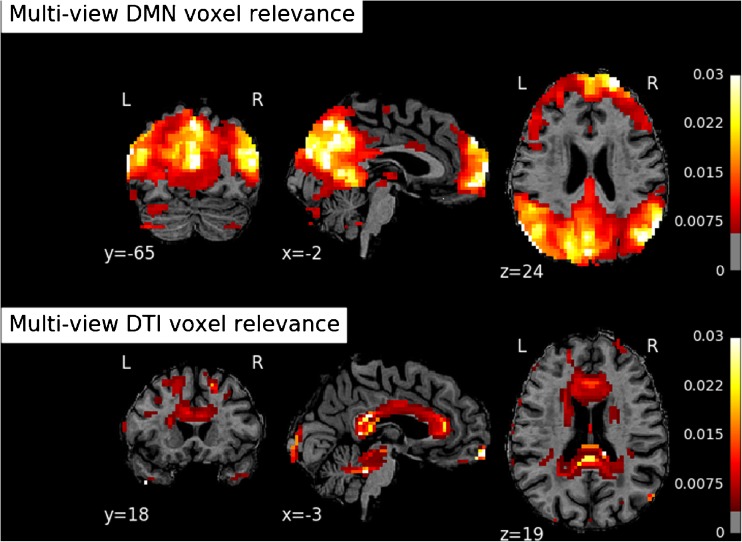
Voxel cluster relevance maps (triplanar reslicing: coronal, sagittal, transversal) computed from the multi-view intermediate integration Random Forest. Upper row: DMN view; Bottom row: FA view

Lastly, we also report the most relevant features in the learned RF models. These can in principle be different between single and intermediate MV models due to a possible effect of data integration on the relative importance of features; this is not the case for late integration models since they were based on SV feature relevance. In Figs. 7 and 8 we highlight the most important clusters of the parcelation for the 3-class discriminations given by the SV and MV models respectively. For both figures, a transparency level is assigned to each cluster of voxels in its entirety. The more relevant a cluster is, the less transparent it is represented. These brain maps suggest that the patterns of relative importance are very similar between SV and MV models. The relevant areas resulting from the SV model trained on the DMN correspond well to the centres of the anchor node regions of the DMN in the medial prefrontal cortex and in the precuneus, with more peripheral regions showing gradually lower importance for the discrimination. For the DTI FA maps resulting from the SV model, the importance of the two cross-hemispheric callosal bundles is evident, with gradually lower importance along the main association bundles that connect the brain from the corpus callosum anteriorly and posteriorly towards the cingulate cortex.

## Discussion

We proposed two novel MV data integration models for RF-based ensemble classification of brain connectivity images from different MRI modalities. They showed that the MV analysis of multiple views can improve the predictive power of individual classifications based on single-subject data.

In general, ensemble classifiers offer a higher margin of accuracy compared to single classifiers. In (Dietterich 2000) three reasons for the advantage of ensemble methods are evidenced: (i) from the statistical viewpoint, when data is scarce compared to its dimensionality, it is easier to find even linear classifiers that perfectly fit the data (overfitting); in contrast an ensemble classifier provides an averaged prediction reducing the generalization error (less overfitting); (ii) computationally, ensemble models that converge to different local minima of the objective criterion (e.g. a decision tree or a neural network) provide a better approximation of the classification function compared to a single model; (iii) if the ideal classification function is not well represented by the functional family of the chosen classifier (e.g. linear SVMs cannot learn non-linear decision functions), as it is the case in many real world applications, by the sole combination of different classifiers, it would extend the class of classification functions and better approximate the ideal classification function.

The brain images chosen as views corresponded to functional and anatomical connectivity maps respectively extracted from rs-fMRI and dw-MRI data sets of the same experimental subjects. In particular, the ICA-derived default-mode network (DMN) component maps and the DTI-derived fractional anisotropy (FA) maps were calculated to provide the algorithms with complementary functional and structural information about large-scale and long-range brain connectivity.

The mean smoothness of the maps corresponding to the two views were different from each other. As we did not apply any spatial smoothing to the data, we fully preserved the original intrinsic smoothness of the calculated maps in the feature agglomeration procedure. In fact, although we expect that applying a spatial smoothing filter prior to this step would increase the smoothness of the maps and therefore produce more compact clusters of voxels with similar variability across subjects, this would also reduce the inter-individual variability. As we are ultimately aiming at classifying individual (multi-view) patterns, we preferred to retain as much as possible of the inter-individual variability of the data, even at the cost of producing less compact clusters at the feature agglomeration stage. In addition, given the different spatial structure of the two views, it would be difficult to anticipate the optimal kernel for each view. Future extensions of the presented methodology may entail with determining the optimal size and the influence of the application of a spatial smoothing filter on the performances of the single- and multi-view classifications.

To reduce the input data set dimensionality, the voxel-wise images from training subjects of each view were initially submitted to a clustering algorithm using a feature (voxel) agglomeration unsupervised procedure (Jenatton et al. 2011) that retained as much information as possible about the inter-subject variability. We did not explicitly enforce anatomical constraints like symmetry between the hemispheres and, in fact, the resulting parcelation is not expected to be anatomically plausible. Nonetheless, this data-driven parcelation has been chosen because it allowed a class-independent dimension reduction without discarding voxels in advance (like e.g. through the application of voxel-level statistical thresholds or anatomical masks from standard atlases (Mwangi et al. 2014)), thereby retaining as much as possible information of the multivariate patterns in the data (Kriegeskorte et al. 2007). Particularly, our feature selection method did not require the performance of group-level statistical tests, even if this could have been computationally attractive. It has been in fact highlighted that feature selection by group comparison is not the optimal method for selecting informative features in machine learning applications because high statistical significances do not imply high classification accuracy (Arbabshirani et al. 2015).

Following (Pavlidis et al. 2001), we explored two different strategies to integrate DMN and DTI views: (i) Intermediate integration, when we performed integration contextually during learning of the classification rule, resulting in a single MV prediction; (ii) Late integration in which we trained two SV RFs separately and then aggregated the resulting SV predictions into one final MV prediction. Due to both anatomical and dimensionality issues, we considered the strategy of early integration not appropriate for our case because the fusion of the two views should have been performed at the voxel level despite fMRI and DTI views are known to carry complementary information in different regions of the brain, made up, respectively, of mainly gray matter and mainly white matter voxels. This aspect was easily confirmed by visually inspecting the voxel importance maps resulting from the trained RF models.

We compared SV and MV data models on the problem of discriminating 121 subjects (HC: 43, ALS: 41, PD: 37) on the basis of their individual DMN and FA maps using the RF classifier. Compared to linear classifiers, such as, e.g., linear support vector machines (SVMs), ensemble models like RFs, are expected to be more robust to noise in high-dimensional settings and easier to train, with less effort on parameter tuning (Kuncheva and Rodríguez 2010). Moreover, RFs can be easily extended to work in MV contexts (Gray et al. 2013).

We also performed some preliminary tests using SVMs for the single views and then using both a majority vote approach (for late integration) and a mixture of linear kernels computed from the DTI and DMN views as an alternative solution for intermediate integration via so called multiple kernel learning or (MKL) (Gönen and Alpaydın 2011). In both cases, the multi-view results were not better (41% Majority Vote, 36.7% mixture of kernels) than the corresponding single-view SVMs (43.4% DMN and 37.3% DTI). For these tests, due to computational reasons, we kept the number of clusters fixed to 500 for both views. Moreover, the parameter C of each SVM and the mixture parameter of the MKL were estimated in nested cross-validation, i.e. the performances of each parameter set were estimated with an inner leave-one-out cross-validation and then the generalization performances of the best set of parameters were estimated with an outer 5-fold cross-validation. We believe that the lower performances observed for MKL may be due to the relatively low amount of complementary information between the views and/or to the low number of observations available (patients). Indeed, a systematic review of four data integration methods applied to linear SVMs (Pettersson-Yeo et al. 2014) has previously shown that MKL does not always perform better than simpler integration methods (like unweighted kernel averaging or majority vote) in cases where the views carry relatively more common (and less complimentary) information and for small sample sizes. On the other hand, the sample size used in the present work is very common in neuroimaging studies. As we only considered linear SVMs, further investigations are still needed for the exploration of non-linear kernels, even though these are also not usual in neuroimaging due to so called “pre-image” problem that anyway limits the interpretation of the estimated models (Kwok and Tsang 2004).

Results of RF models showed that the proposed MV classification methods yield, on average, improved accuracy compared to SV classifiers built on DMN or FA maps separately. This suggests that mining complementary information from the integration of multiple data views may lead to a better understanding of the phenomena under study.

The improvement of the proposed MV vs. SV models was more evident for the ALS class where both intermediate and late integration models achieved a higher classification accuracy compared to both SV models. Class-specific classification improvements were observed for the PD class where both intermediate and late integration models reached an accuracy comparable to the best SV result (the DMN view in this case) and better than the worse SV result (the DTI view in this case). Similar trends were seen for the HC class, but, compared to patients; the overall classification accuracies of HCs reached lower values in both SV and MV data models. This might be due to bad performances of the RF classifiers (due to, e.g., poor representativeness of the data sets or small sample size), to the higher general difficulty of correctly classifying elderly HCs with (only) the chosen views, or to a combination of both. As a matter of fact, it has been sometimes reported that physiological age-related changes of functional connectivity of resting state BOLD signals in healthy populations are not only characterized by possible reductions in the resting-state functional connectivity within the DMN (similar to various clinical populations) but also by ubiquitous increases in internetwork correlations, i.e. functional connectivity effects that involve connections between the DMN and other attentional networks (Lustig et al. 2003; Damoiseaux et al. 2008; Ferreira and Busatto 2013), with the consequence that the DMN view alone would have more limited capabilities in the separation of HCs from non-HC (e. g. ALS and PD) populations. Future work extending the presented MV framework to more (functional) views will possibly address this aspect directly.

On the other hand, it remains possible that the selected neuroimaging features target some specific patterns of (age-related) neurodegeneration that are not exclusively limited to pathological conditions but rather affect with similar modalities the healthy like the pathological brains. In this case, we would expect that possible misclassifications of HCs would not result as random errors of the RF classifiers but as consistent wrong assignments of a subject to one specific pathological class. To address this question, we further explored the possibility that some HCs would be systematically, and not randomly, classified as ALS or PD. However, to rule out that RF classification errors were due to an overall poorly representative training set, we created *100* random subsets of training samples and repeated *100* times the training of each RF model, separately for each single HC, which was always excluded from all training sets. We found that the misclassification of some HCs as PD or ALS was highly consistent with one of the two pathological classes and stable across all training sets. In an additional experiment, we also repeated the classification of all patterns using RF models trained only on patients’ data sets. In this case, the models were completely missing the information from control patterns and could only assign each pattern to the ALS or PD class. As expected, the accuracies of both SV and MV classifiers considerably increase for patients and both MV classifiers outperformed at least one of the two SV classifiers. For HCs, systematic misclassifications in the 3-class problem were further confirmed in the 2-class problem, i.e. HCs systematically assigned to the same (ALS or PD) class by both the 3-class and the 2-class RF models. Thereby, we cannot definitely exclude that the lower discriminative power of the original 3-class RF classifiers observed in our data is at least in part due to the fact that RF models can capture some specific effects of physiological neurodegeneration (probably, albeit not necessarily, associated with aging) because of a connectivity pattern shared by some HCs with either PD or ALS patients and mimicking an ALS-type or PD-type neurodegeneration process.

Both SV and MV RF models provide insight of how relevant each feature is for the classification as a by-product of training. Since we used brain areas as features, we derived a set of maps which could be visually inspected to verify the plausibility of the maps. The feature importance maps appeared very similar between SV and MV models and corresponded well to the main node regions of the DMN in the grey matter and the main association bundles in the white matter, suggesting that the chosen clustering technique for voxel aggregation retained the relevant pattern information in the reduced set of features.

Finally, to rule out whether the ALS patients had a significant weight on the feature agglomeration, we also performed a single-class agglomeration of features, however, the resulting parcelation did not show noticeable differences in the resulting clusters among classes, suggesting that the feature agglomeration mainly captures general class-independent similarities of voxels in their variations across subjects. In sum, we believe that the differences among groups are encoded in subtle differences among whole-brain distributed patterns, possibly due to feature values derived by a more general parcelation of the brain that cannot be easily recognizable by visual inspection.

Although the results of integrating structural and functional connectivity of the same subjects are promising, this application suffers from some limitations. First, as for most Machine Learning applications to neuroimaging problems, the sample size is a limiting factor. Although our sample size is near the median of sample sizes of similar studies published during the last few years, our approach as well as other similar methods proposed, may greatly benefit from larger sample sizes, in which the heterogeneity of diseases could be also better characterized (Arbabshirani et al. 2015). Second, the spatial scale of the parcelation was determined for each imaging view separately by cross-validation. An adaptive method able to determine the best suitable parcelation given the data may lead to more robust and discriminative features. The supervised cut procedure proposed in (Michel et al. 2012) is an example in this direction, even though there is no guarantee for optimal performances. Lastly, the relevant features returned by the models only bring information on which areas are the most relevant to the classification accuracy, but do not provide information about which area is the most discriminative for a given class, neither provide information about the characterization of a class in terms of the values of the features. We hypothesize that a custom procedure to evaluate the criterion of mean accuracy decrease (see, e. g.,(Archer and Kimes 2008)) could be developed to obtain a set of group-specific relevant areas.

Future work is also needed to adapt the proposed multi-view classification framework to work with classical connectivity measures (e.g. correlation or covariance) for the functional view. In fact, as ICA values do not measure the connectivity between two specific regions, they would not be reliable for subject classification if the set of regions is defined a priori, from independently determined brain networks or standard atlases, as required by classical connectivity measures, but rather need to be used in combination with some form of data-driven voxel clustering, as proposed here.

In conclusion, we proposed two MV data models to integrate functional and structural connectivity maps for the same subjects to be used with an RF classifier to classify ALS and PD patients as well as HC. As features, we used voxel clusters derived by aggregating contiguous voxels in an unsupervised fashion. The trained data models were based on the RF framework that has been extended to work with MV data sets. Accuracy performances were comparable or higher for MV compared to the SV data models, although there were differences among the classes. We further investigated the prediction accuracy in HCs and hypothesized that the used features may encode aspects of neurodegeneration that can be sometimes similar between physiological and different types of pathological aging, making it more difficult the discrimination of some HCs as HCs. In future works, the same approach can be used to classify images of different brain networks as alternative or additional views and the entire MV framework can be further extended to combine imaging with non-imaging views, such as clinical, behavioural or even genetic multidimensional data, when available from the same subjects.

## Information Sharing Statement

Source code (python scripts) implementing the methods and the analyses described in this paper can be requested to Michele Fratello at michele.fratello@unina2.it.
